# SCGB3A1‐Epi and KLK10‐Epi Crosstalk With Fibroblasts Promotes Liver Metastasis of Breast Cancer and Pancreatic Ductal Adenocarcinoma

**DOI:** 10.1002/cam4.70904

**Published:** 2025-05-13

**Authors:** Zixue Xuan, Zhongxiu Wu, Lei Cheng, Jinying Jiang, Yuan Zhang, Yuxuan Xia

**Affiliations:** ^1^ Center for Clinical Pharmacy, Cancer Center, Department of Pharmacy, Zhejiang Provincial People's Hospital (Affiliated People's Hospital) Hangzhou Medical College Hangzhou Zhejiang China; ^2^ Department of Pharmacy Zhejiang Provincial People's Hospital Bijie Hospital Bijie Guizhou China; ^3^ Outpatient Department, Zhejiang Provincial People's Hospital (Affiliated People's Hospital) Hangzhou Medical College Hangzhou Zhejiang China

**Keywords:** breast cancer, epithelial cell subtypes, liver metastasis, pancreatic ductal adenocarcinoma, single‐cell RNA sequencing, tumor microenvironment

## Abstract

**Background:**

The liver often serves as the principal site for metastatic spread from a variety of solid tumors, and metastasis to the liver markedly diminishes patient survival. Single‐cell RNA sequencing (scRNA‐seq) has helped uncover the complexity of liver tumor metastasis. However, the key cellular subtypes of breast cancer and pancreatic ductal adenocarcinoma (PDAC) with liver metastasis and their mechanisms of action are unclear, making treatment difficult.

**Methods:**

We used integrated scRNA‐seq data to dissect liver metastasis‐specific epithelial cell subtypes in breast cancer and PDAC, and elucidated their mechanisms through functional analyses and intercellular interactions with fibroblasts.

**Results:**

Interestingly, our results show that SCGB3A1‐Epi and KLK10‐Epi are key drivers of liver metastasis in breast cancer and PDAC, respectively. These subtypes are associated with high malignancy rates and involved in oxidative phosphorylation and other critical pathways. Specific ligand‐receptor interactions were observed between these epithelial subtypes and fibroblasts, with significant interactions between CD74‐APP receptors in SCGB3A1‐Epi and Fib‐11 in breast cancer and between SPP1‐CD44 receptors in KLK10‐Epi and Fib‐11 in PDAC. High expression levels of Fib‐11 and CD74 were correlated with improved survival in breast cancer, whereas high SPP1 and CD44 expression predicted worse PDAC outcomes. Fib‐11 is implicated in signaling pathways associated with tumor metastasis, particularly those involving cell adhesion molecules.

**Conclusions:**

We revealed the cellular heterogeneity of liver metastasis and provided a crucial research foundation for developing novel therapeutic strategies to specifically target metastatic cell subtypes, thereby enhancing patient prognosis.

**Abbreviations**
scRNA‐seq: single‐cell sequencing
PDAC: pancreatic ductal adenocarcinoma
CNV: copy number variation
HCC: hepatocellular carcinoma
LM: liver metastasis
LM‐CC: the liver metastasis of colorectal cancer
LM‐BC: liver metastasis of breast cancer
LM‐PDAC: liver metastasis of PDAC
DEG: differentially expressed gene
TCGA: The Cancer Genome Atlas

## 1 Introduction

Cancer metastasis poses a formidable global public health challenge and is noted for its heterogeneity and high fatality, with the management of metastatic tumors continuing to elude easy solutions [1]. In recent years, tumor cell heterogeneity has emerged as a crucial focus in cancer research. For instance, Grinda et al. discovered substantial phenotypic discordance between primary breast tumors and metastatic sites through single‐cell RNA sequencing (scRNA‐seq) analysis, and the phenotypic discordance may profoundly influence the selection of therapeutic strategies [2]. Additionally, Sanjaya et al. employed scRNA‐seq to elucidate the heterogeneity of breast cancer metastasis, revealing that tumor cells at different metastatic sites exhibit distinct gene expression profiles and potential biological behaviors [3]. Colorectal cancer often demonstrates polyphyletic seeding, a phenomenon that can result in late metastases characterized by significant inter‐metastatic driver gene heterogeneity [4]. These studies underscore the pivotal role of tumor cell heterogeneity in cancer metastasis, highlighting that tumor cells at different metastatic sites may display diverse phenotypes and functions.

Organ‐specific metastasis, or organ tropism, is a hallmark of the spread of cancer [5]. Breast cancer, for example, frequently metastasizes to the lungs, liver, bones, and brain [6], whereas prostate cancer exhibits a pronounced inclination toward bone metastasis [7]. The liver is a particularly common site for metastasis, attracting various cancers including those of the breast, colorectum, pancreas, stomach, lungs, and ovaries [8, 9]. This predilection is partly attributed to the rich vascular network of the liver, which renders it a favored destination for metastatic tumor cells [10]. Additionally, tumor cells may express specific molecular markers, including adhesion molecules and growth factor receptors, which facilitate their interaction with the hepatic microenvironment, thereby promoting metastasis and colonization [11, 12]. Research has highlighted how the interaction between the tumor‐surface receptor ROBO1 and the hepatocyte‐derived ligand SLIT2 fosters an adaptive dialog between tumor cells and hepatocytes, contributing to liver metastasis of pancreatic ductal adenocarcinoma (PDAC) [13]. Moreover, cell clusters migrating from the primary tumor appear to be more efficacious in establishing distant metastases than individual tumor cells, underscoring the pivotal role of specific cell subtypes in tumor metastasis [14, 15, 16]. In our prior studies, we identified that Epi‐11 cells, a significant subset of epithelial cells, potentially propel liver metastasis in colorectal cancer through interactions with immune cells via the PLXNB1/SEMA4D signaling axis [12]. Nonetheless, the liver metastasis of tumors is enshrouded in complex molecular mechanisms and tumor microenvironment interactions, with the possibility that distinct primary tumors harbor unique driver cells for liver metastasis [17]. Moreover, evidence suggests that both direct cell‐to‐cell contact and indirect communication via secreted factors between fibroblasts and tumor cells can exacerbate tumor progression and metastasis [18]. However, the key cellular subtypes of colorectal cancer, breast cancer, and PDAC that metastasize to the liver and their mechanisms of action are still unclear, leading to difficulty in the treatment of patients with liver metastasis [19, 20, 21]. Therefore, this study aims to integrate and analyze scRNA‐seq data to investigate the cellular heterogeneity of liver metastasis, screen for potential driver cell subtypes of liver metastasis, and preliminarily reveal their interaction with fibroblasts in liver metastasis. The results of this study will provide an important research foundation for specifically inhibiting cellular subtypes with metastatic potential, effectively controlling tumor liver metastasis, which is important for developing new therapeutic strategies and improving patient prognosis.

## 2 Methods

### 2.1 Data Source

In this study, 34 samples from 26 patients were analyzed. The samples included eight non‐cancerous liver tissues adjacent to hepatocellular carcinoma (from GSE149614), 10 primary hepatocellular carcinoma (HCC) samples (from GSE149614) [22], and 16 liver metastatic samples. Among the liver metastatic samples, three were from colorectal cancer (from GSE178318; only liver metastatic samples from untreated patients with colorectal cancer were included) [23], six were from breast cancer (from GSE249361) [24], and seven were from PDAC (from GSE263733) [25].

### 2.2 scRNA‐Seq Analysis

To filter out low‐quality cells and doublets for each sample, the cells that had over 6000 or below 200 expressed genes were removed. To filter out dead or dying cells, the cells that had over 5% mitochondrial genes were further removed. Then, cells were finally obtained for subsequent analysis. We employed the Harmony (version 3.8) method to perform batch corrections on each dataset. Subsequently, we utilized the FindNeighbors function and FindClusters function from the Seurat (version 5.01) to group cells into distinct clusters, thereby identifying different cell types or subpopulations. The clustering results were visualized using the UMAP dimensionality reduction technique [26]. To identify primary cell types, we referred to established cell markers from the original articles [22, 23, 24, 25]. To illustrate the diversity and complexity of cell types within the samples, we used the Wilcoxon rank‐sum test to assess differences in cell proportions among groups, with *p* < 0.05 considered statistically significant [27].

### 2.3 Identification of Epithelial Cell Subtypes That Drive Liver Metastasis

Via the Seurat RunPCA, RunUMAP, and Findclusters functions, we performed epithelial cell subtypes clustering [26]. The proportion of each epithelial subset within the total epithelial cell population was calculated for each sample, following the same statistical method used for comparing the main cell types [27]. Furthermore, we used inferCNV (version 1.17.0) to assess copy number variation (CNV) levels across all cells, with a specific focus on epithelial cell subtypes [12].

### 2.4 Functional Analysis of Epithelial Cell Subtypes

We analyzed the differentially expressed genes (DEGs) within the cellular subtypes using the FindMarkers function in Seurat (version 5.01), with parameters aligned with our previous analyses [12]. In this study, the DEGs were identified by comparing the analyzed subcluster against the remaining epithelial subclusters. Next, we performed enrichment analysis of these characteristic genes against hallmark gene sets and KEGG pathways, with *p* < 0.05 considered statistically enriched [28].

### 2.5 Intercellular Interactions Between Epithelial Cell Subtypes and Fibroblasts

We performed clustering and visualization of scRNA‐seq data using the Seurat RunPCA, RunUMAP, and Findclusters functions to annotate the fibroblast subtypes [26]. CellPhoneDB (version 5.0.0) was used to analyze intercellular receptor–ligand interactions between epithelial cell subtypes and fibroblasts [29, 30].

### 2.6 Prognostic Correlation Analysis

Based on the expression profiles of characteristic genes from epithelial cells, fibroblast subtypes, or characteristic molecules, we integrated these data with clinical information from The Cancer Genome Atlas (TCGA) to analyze the correlation between these cellular subtypes or molecular features and patient prognosis. Specifically, we employed the Survfit function from the R package survival to analyze these correlations [31].

## 3 Results

### 3.1 Single‐Cell Transcriptomic Profiling of Liver Metastases From Colorectal Cancer, Breast Cancer, and PDAC

**FIGURE 1:**
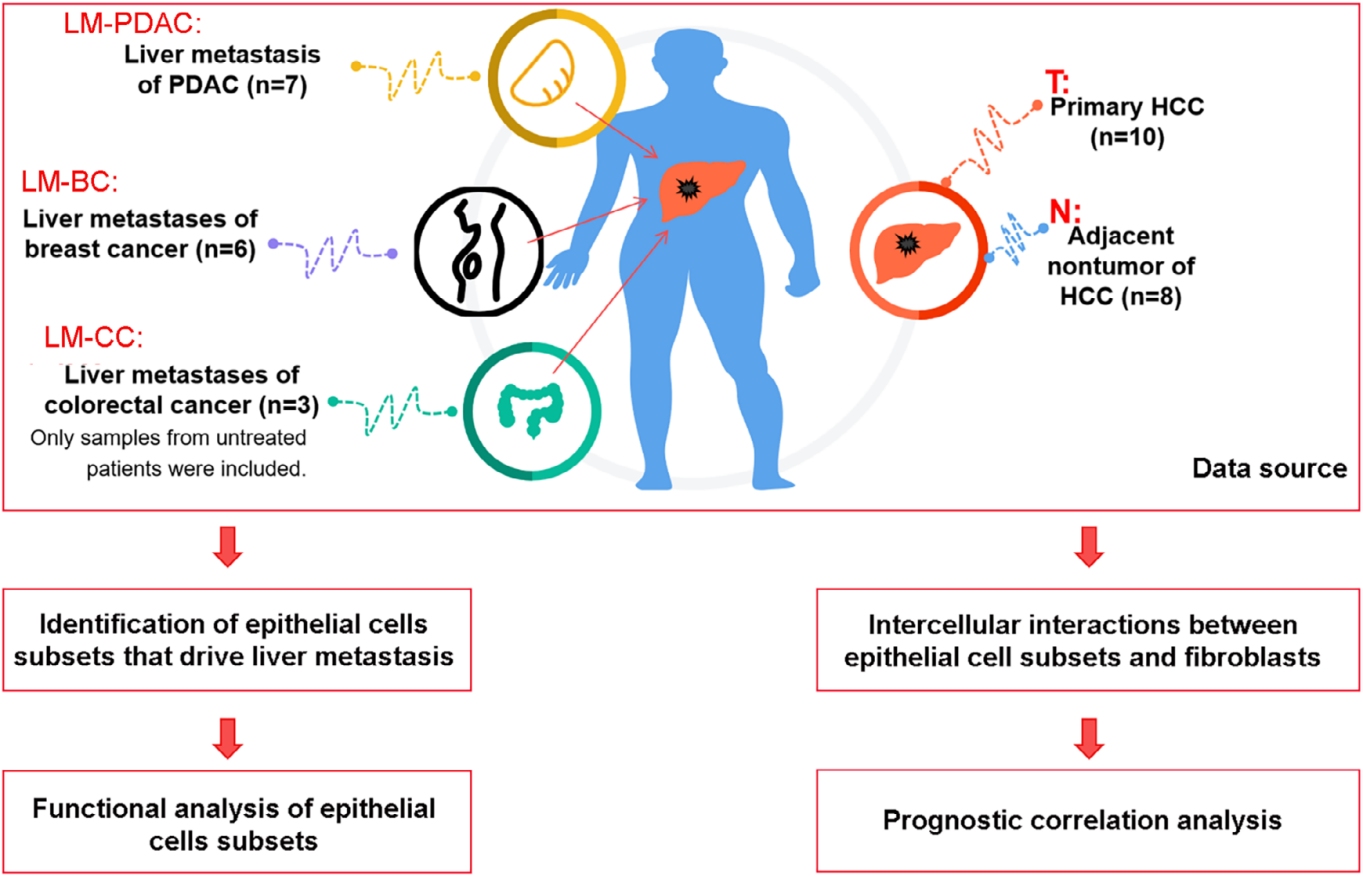
The workflow and detailed sample information.

We conducted a comprehensive single‐cell transcriptomic analysis of liver metastases from colorectal cancer, breast cancer, PDAC, primary HCC, and adjacent non‐tumor tissues. The workflow and detailed sample information are shown in Figure 1A.

**FIGURE 2:**
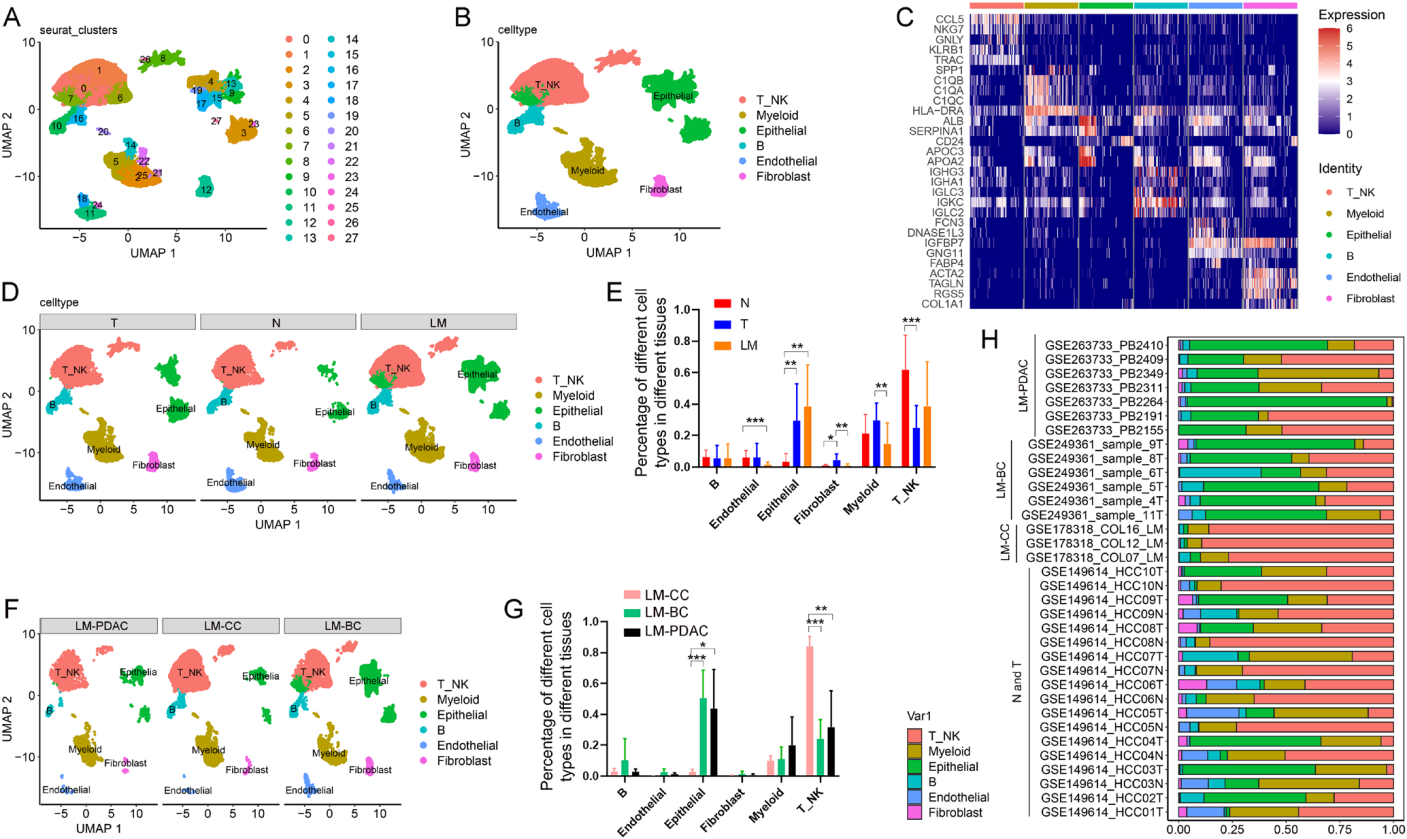
Single‐cell transcriptomic profiling of liver metastases from colorectal cancer, breast cancer, and PDAC. (A) UMAP visualization of 28 cell clusters. (B) UMAP visualization of T_NK cells, myeloid cells, epithelial cells, B cells, endothelial cells, and fibroblasts. (C) The heatmap showing the expression levels of established markers for distinct cellular clusters. (D) UMAP visualization of 6 cell types in T, N, and LM groups. (E) Number of 6 cell types in T, N, and LM groups. (F) UMAP visualization of 6 cell types in different liver metastatic samples. (G) Number of 6 cell types in LM‐CC, LM‐BC, and LM‐PDAC groups. (H) Distribution of cell types in all samples. *, *p* < 0.05; **, *p* < 0.01; ***, *p* < 0.001.

Following rigorous quality control and batch effect correction, we analyzed the scRNA‐seq data from 94,384 cells (Figure 2A). By employing established markers for distinct cellular clusters, we identified six major cell types: T_NK cells (T cells or NK cells), myeloid cells, epithelial cells, B cells, endothelial cells, and fibroblasts (Figure 2B,C). The relative abundances of these cell types across various tissue samples are illustrated in Figure 2D. We observed no significant variation in B‐cell proportions among the adjacent non‐tumor tissue (N), primary HCC (T), and liver metastasis (LM) groups (LM vs. N, *p* = 0.8458; LM vs. T, *p* = 0.9777). Endothelial cells were significantly reduced in the LM group compared to the N group (*p* = 0.001). Epithelial cell proportions were markedly increased in the T and LM groups compared to the N group (LM vs. N, *p* = 0.0012; T vs. N, *p* = 0.0075), whereas T and NK cell proportions were significantly decreased in the T group (LM vs. N, *p* = 0.0557; T vs. N, *p* = 0.0006). Fibroblasts were more abundant in the T group than in the N or LM groups (T vs. N, *p* = 0.041; T vs. LM, *p* = 0.0044). Myeloid cells were less abundant in the LM group than in the T group (*p* = 0.0064) (Figure 2E).

Further analyses revealed that among the liver metastasis of colorectal cancer (LM‐CC), liver metastasis of breast cancer (LM‐BC), and liver metastasis of PDAC (LM‐PDAC) groups, LM‐BC and LM‐PDAC had the highest proportions of epithelial cells (Figure 2F–H). Additionally, in our recent study, we have specifically investigated the epithelial cells driving liver metastasis in colorectal cancer, identifying the Epi‐11 cells [12]. Consequently, this study focused on the epithelial cells within LM‐BC and LM‐PDAC for detailed analyses.

### 3.2 SCGB3A1‐Epi and KLK10‐Epi Are the Epithelial Cell Subtypes Driving Liver Metastasis of Breast Cancer and PDAC, Respectively

**FIGURE 3:**
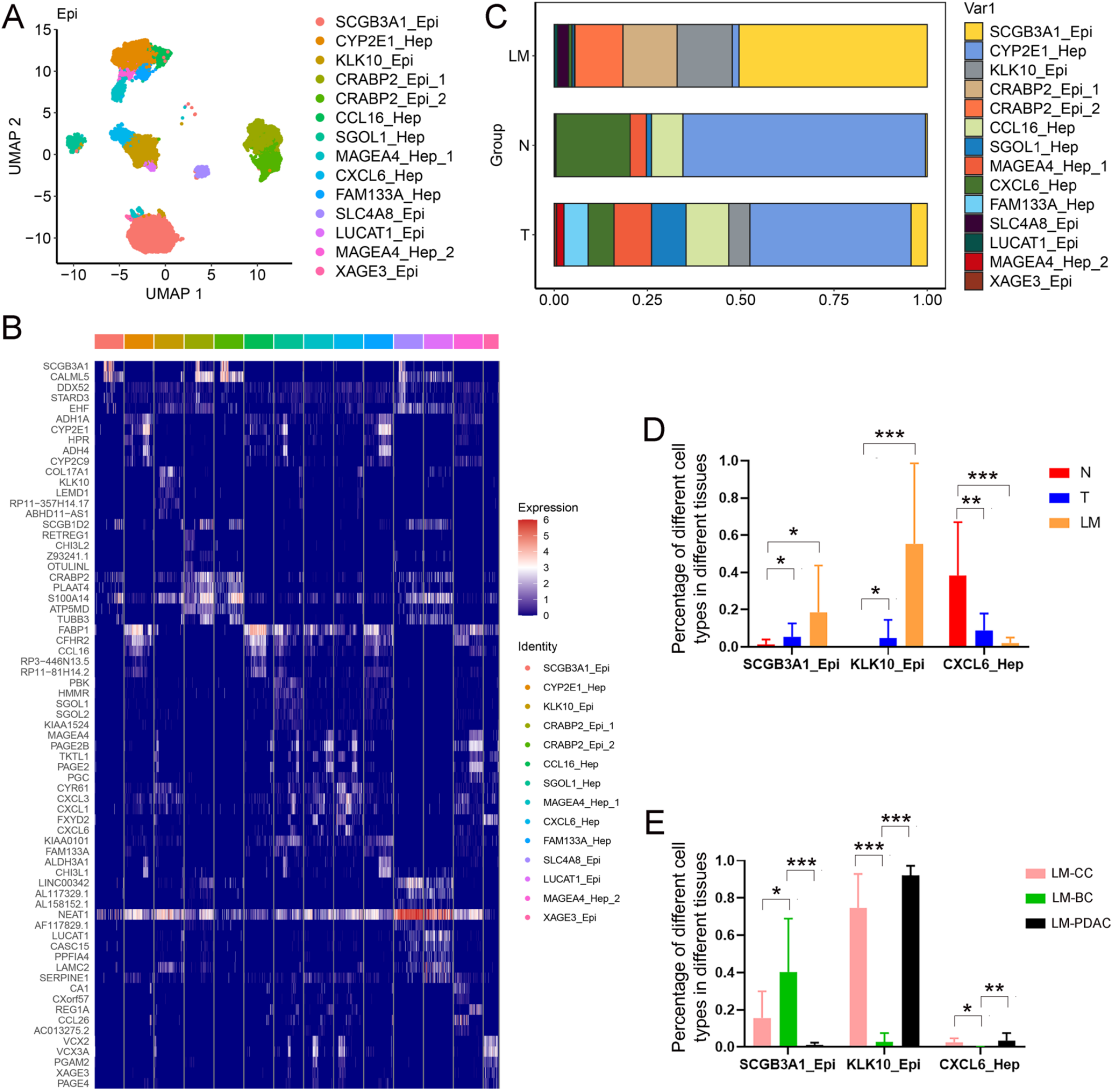
The level of SCGB3A1‐Epi and KLK10‐Epi increases in LM groups. (A) UMAP visualization of 14 cell subtypes in epithelial cells. (B) The heatmap showing the expression levels of markers for 14 cell subtypes. (C) Relative proportions of 14 cell subtypes in T, N, and LM groups. (D) Number of SCGB3A1‐Epi, KLK10‐Epi, and CXCL6‐Hep in T, N, and LM groups. (E) Number of SCGB3A1‐Epi, KLK10‐Epi, and CXCL6‐Hep in LM‐CC, LM‐BC, and LM‐PDAC groups. *, *p* < 0.05; **, *p* < 0.01; ***, *p* < 0.001.

We conducted a sub‐classification of epithelial cells and identified 14 distinct cell subtypes in all patients (Figure 3A); their marker genes are detailed in Figure 3B. We compared the expression of SCGB3A1‐Epi and KLK10‐Epi in the T and LM groups relative to that in the N group and revealed a significant increase in the proportion of SCGB3A1‐Epi and KLK10‐Epi cells in both the T and LM groups (Figure 3C,D). Notably, SCGB3A1‐Epi was enriched in the LM‐BC group, whereas KLK10‐Epi was enriched in the LM‐CC and LM‐PDAC groups (Figure 3E). Conversely, CXCL6‐Hep expression significantly reduced in the T and LM groups (Figure 3D).

**FIGURE 4:**
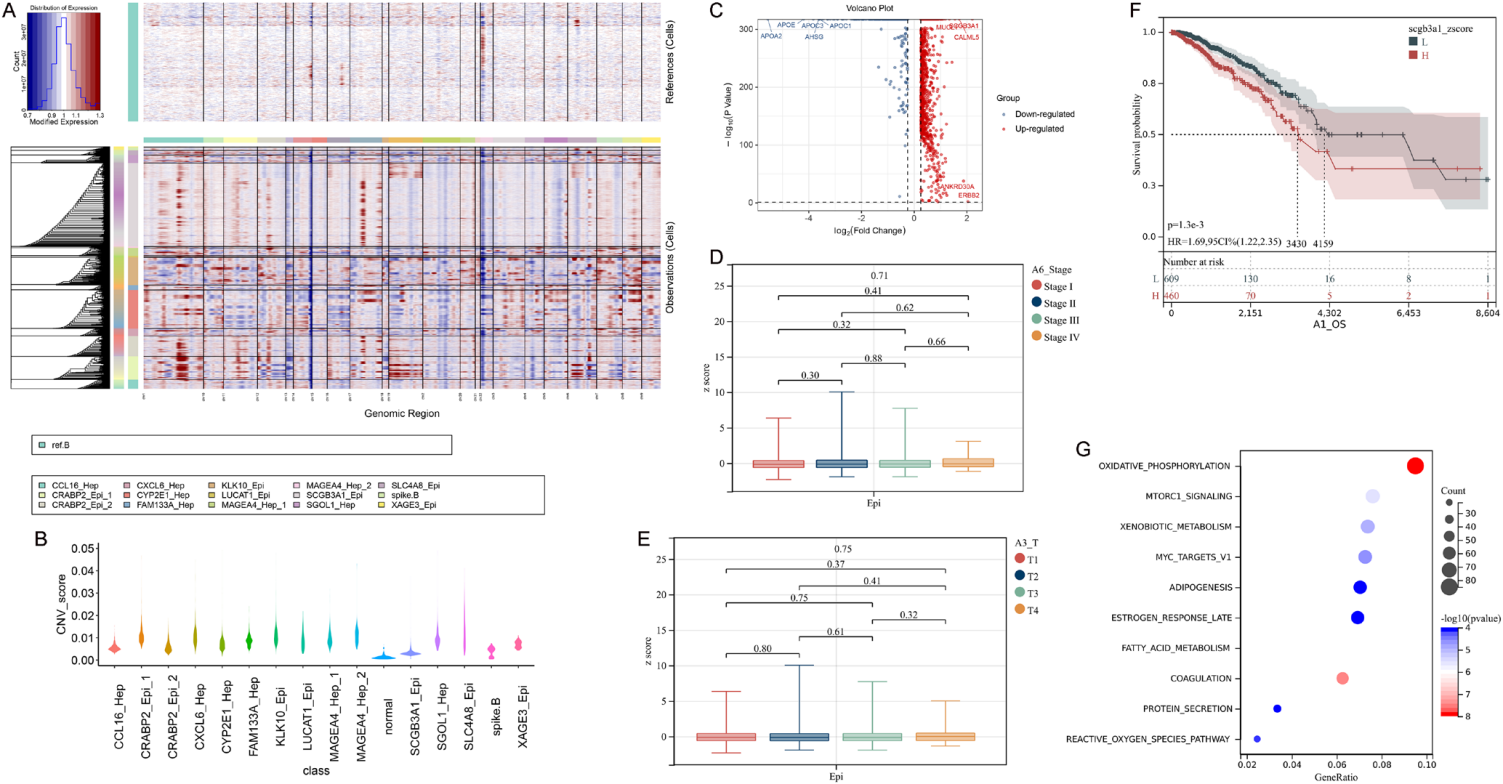
SCGB3A1‐Epi are the epithelial cell subtypes driving liver metastasis of breast cancer. (A) InferCNV's analysis of 14 cell subtypes in epithelial cells. (B) SCGB3A1‐Epi and KLK10‐Epi exhibited significantly higher CNV scores. (C) The DEGs of SCGB3A1‐Epi. (D–F) The correlation of SCGB3A1 expression with the stage, the T‐stage, and the overall survival of breast cancer patients, respectively. (G) Functional enrichment analysis of total DEGs in SCGB3A1‐Epi.

SCGB3A1‐Epi and KLK10‐Epi cells exhibited significantly higher CNV scores (Figure 4A,B), suggesting that both SCGB3A1‐Epi and KLK10‐Epi cells had a high degree of malignancy. We screened the differentially expressed genes (DEGs) of SCGB3A1‐Epi (Figure 4C). In this study, the DEGs were identified by comparing the analyzed subcluster against the remaining epithelial subclusters. We then analyzed the correlation between SCGB3A1 expression and the clinical characteristics of patients with breast cancer and found no significant association with stage or T stage (Figure 4D,E). Kaplan–Meier survival analysis indicated that patients with breast cancer with elevated SCGB3A1 levels had significantly shorter survival times (Figure 4F). Functional enrichment analysis of total DEGs with FDR < 0.1 and *p* < 0.05 revealed associations with pathways such as oxidative phosphorylation, coagulation, fatty acid metabolism, mTORC1 signaling, xenobiotic metabolism, myc targets v1, reactive oxygen species pathway, adipogenesis, estrogen response late, protein secretion, mitotic spindle, and cholesterol homeostasis (Figure 4G).

**FIGURE 5:**
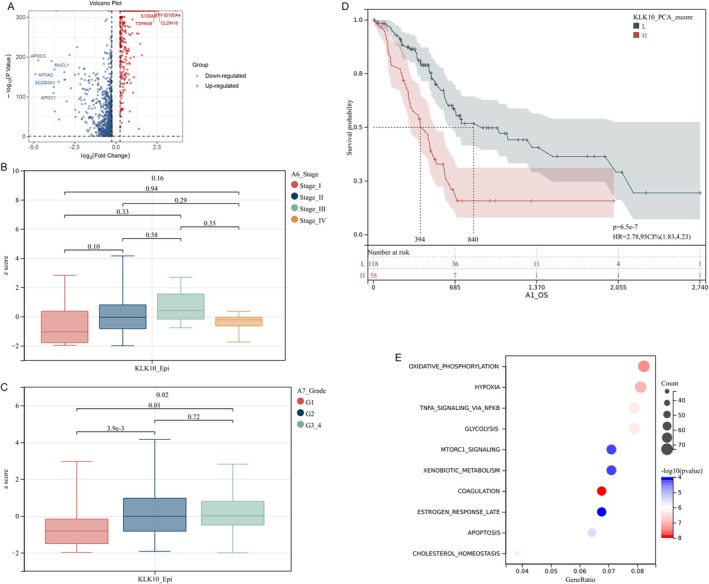
KLK10‐Epi are the epithelial cell subtypes driving liver metastasis of PDAC. (A) The DEGs of KLK10‐Epi. (B–D) The correlation of KLK10 expression with the stage, the grade, and the overall survival of PDAC patients, respectively. (E) Functional enrichment analysis of total DEGs in KLK10‐Epi.

Similarly, we screened the DEGs of KLK10‐Epi (Figure 5A) and analyzed their correlation with the clinical features of patients with PDAC. We found that both the grade 2 (G2) and G3/G4 groups had higher KLK10‐Epi scores than the G1 group (Figure 5B,C). The Kaplan–Meier survival analysis showed that patients with PDAC with high KLK10 levels had significantly shorter survival times (Figure 5D). The DEGs in KLK10‐Epi were associated with pathways including coagulation, oxidative phosphorylation, hypoxia, cholesterol homeostasis, tnf signaling via NFkb, glycolysis, apoptosis, mTORC1 signaling, xenobiotic metabolism, and estrogen response late (Figure 5E). Comparative analysis revealed that the DEGs in the SCGB3A1‐Epi and KLK10‐Epi groups were involved in the regulation of oxidative phosphorylation, xenobiotic metabolism, and coagulation.

### 3.3 Fibroblasts Interact With SCGB3A1‐Epi and KLK10‐Epi in LM

**FIGURE 6:**
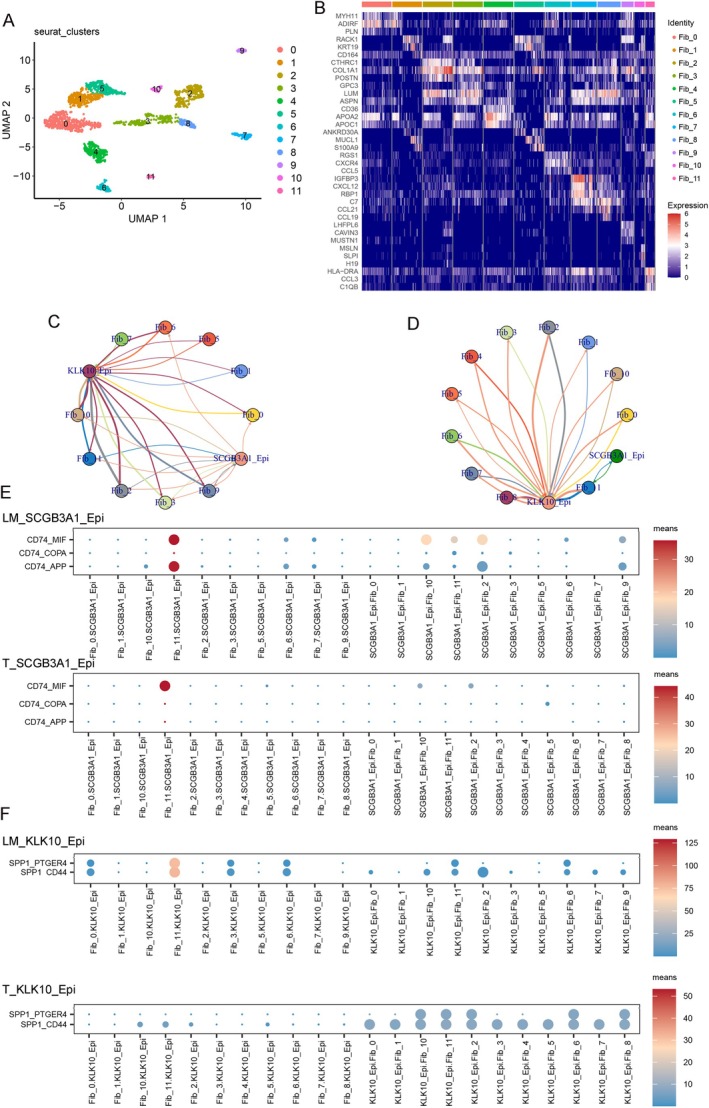
Fibroblast interacts with SCGB3A1‐Epi and KLK10‐Epi in LM. (A) UMAP visualization of 12 cell subtypes in fibroblast. (B) The heatmap shows the expression levels of markers for 12 cell subtypes of fibroblast. (C) The cellphonedb network in LM. (D) The cellphonedb network in T. (E) Bubble heatmap showing the mean interaction strength between SCGB3A1‐Epi and cell subtypes of fibroblast for ligand‐receptor pairs in LM and T. (F) Bubble heatmap showing the mean interaction strength between KLK10‐Epi and cell subtypes of fibroblast for ligand‐receptor pairs in LM and T. Dot size represents the statistical significances as determined by permutation tests, while dot color corresponds to the mean levels of interaction strength.

Cancer metastasis is a complex process influenced not only by cancer cells but also by the tumor microenvironment, including fibroblasts [32, 33]. We identified 12 fibroblast subtypes across all samples based on specific markers (Figure 6A,B) and quantified the strength of ligand–receptor interactions to identify the molecules mediating fibroblast interactions with SCGB3A1‐Epi or KLK10‐Epi, potentially promoting liver metastasis (Figure 6C,D). The CD74‐APP pair was significantly enriched in the LM group between the SCGB3A1‐Epi and Fib‐11 cells, and the SPP1‐CD44 pair was significantly enriched in the LM group between the KLK10‐Epi and Fib‐11 cells (Figure 6E,F).

**FIGURE 7:**
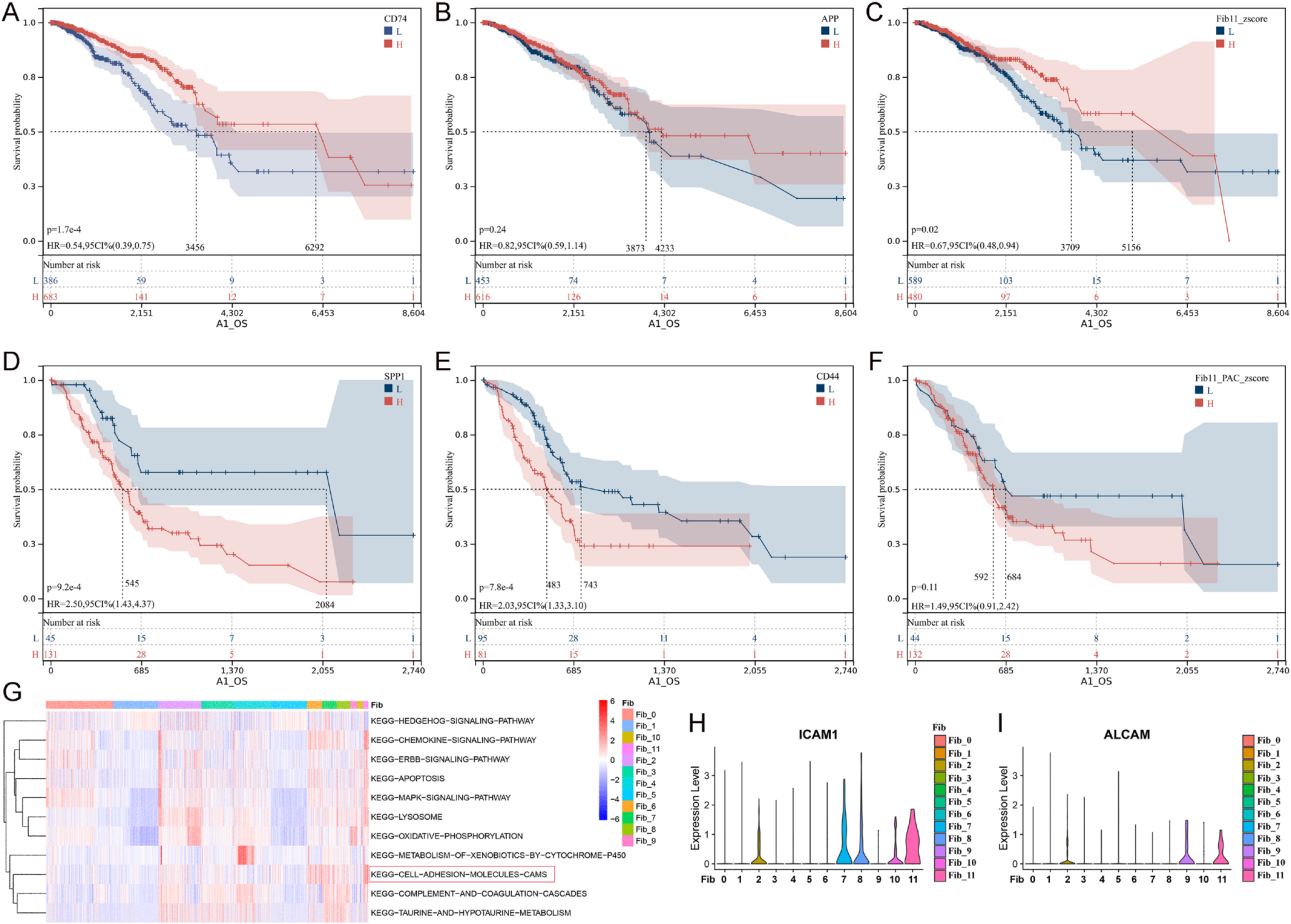
Possible mechanisms of Fib‐11 regulation of liver metastasis in breast cancer or PDAC. (A–C) Kaplan–Meier estimation of survival time of patients with breast cancer by the expression level of CD74, APP, and Fib‐11. (D–F) Kaplan–Meier estimation of survival time of patients with PDAC by the expression level of SPP1, CD44, and Fib‐11. (G) KEGG functional enrichment analysis of total DEGs in Fib‐11. (H, I) The expression level of adhesion molecules ICAM1 and ALCAM in different cell subtypes of fibroblast.

Analysis of the correlation between CD74 and APP and breast cancer patient survival in the TCGA cohort revealed that higher CD74 levels were associated with significantly longer survival times (Figure 7A). However, no significant correlation was observed between the APP levels and survival time (Figure 7B). In PDAC, the survival time was significantly reduced in patients with high levels of SPP1 and CD44 (Figure 7D,E).

A specific relationship between Fib‐11 and SCGB3A1‐Epi or KLK10‐Epi was observed in our ligand–receptor relationship analysis, suggesting that Fib‐11 is an important cellular subgroup involved in liver metastasis. The Kaplan–Meier survival analysis showed that patients with breast cancer with high Fib‐11 levels had significantly longer survival times (Figure 7C), whereas no significant correlation was observed between PDAC patient survival time and Fib‐11 levels (Figure 7F). The KEGG functional enrichment analysis revealed that Fib‐11 is primarily involved in chemokine signaling pathways, lysosomes, oxidative phosphorylation, and cell adhesion molecules, particularly in the latter pathway (Figure 7G). We also found that the adhesion molecules ICAM1 and ALCAM were predominantly expressed in Fib‐11 (Figure 7H,I).

## 4 Discussion and Conclusion

The liver often serves as the principal site for metastatic spread from a variety of solid tumors; liver metastasis markedly diminishes patient survival. The biological activity of liver metastasis is identical to that of the primary tumor, and its treatment is typically determined based on the primary tumor, which differs from the treatment of primary HCC [34]. Current multiomics studies often analyze the cellular heterogeneity of metastatic liver cancer in relation to the primary tumor, with few studies examining the heterogeneity between metastatic liver cancer and HCC [23, 24, 25, 35]. This study focused on the cellular heterogeneity of liver metastatic samples from different tumors, primary HCC, and adjacent normal tissues.

Research has indicated that metastatic liver cancer from various tumors may share common cellular subtypes that drive metastasis to the liver, distinct from those found in HCC [36]. We identified a significant increase in SCGB3A1‐Epi in breast cancer liver metastasis and KLK10‐Epi in PDAC liver metastasis. High expression levels of SCGB3A1 in breast cancer patients and KLK10 in PDAC patients are associated with significantly reduced survival times. These findings suggest that SCGB3A1‐Epi and KLK10‐Epi are the driving cells of liver metastasis in breast cancer and PDAC, respectively.

Previous studies have shown that SCGB3A1 is a tumor suppressor gene, and reduced SCGB3A1 expression is associated with hypermethylation of its promoter in most breast cancer cell lines (> 90%) and primary tumors (74%) [37]. SCGB3A1 hypermethylation is also found in bone, brain, and lung metastases of breast cancer [38]. However, our study found that high SCGB3A1‐Epi expression drives liver metastasis in breast cancer, highlighting the need for further investigation into the function and the underlying mechanisms.

KLK10 is highly expressed in tumors and some immune cells, regulating tumor cell proliferation and differentiation and maintaining tumor function and immune modulation through T cells and macrophages [39]. High KLK10 expression is significantly associated with reduced survival time in patients with colorectal and ovarian cancer [40, 41]. Studies have shown that KLK10 from tumor endothelial cells accelerates colon cancer cell proliferation and hematogenous liver metastasis [42], consistent with our findings that KLK10‐Epi promotes liver metastasis in PDAC.

We then investigated the possible regulatory mechanisms and found that SCGB3A1‐Epi and KLK10‐Epi are involved in the regulation of oxidative phosphorylation, xenobiotic metabolism, and coagulation. Oxidative phosphorylation is a process known to modulate liver metastasis through mechanisms such as the reprogramming of mitochondrial oxidative phosphorylation in tumor cells and impairment of immune cell functions within the tumor microenvironment, contributing to immune evasion [43, 44]. Xenobiotic metabolism plays a crucial role in tumorigenesis and progression through the transcriptional regulation of cytochrome P450 enzymes and the modulation of microRNAs [45]. Coagulation markers have emerged as independent predictors of aggressiveness in prostate cancer and pancreatic cancer [46, 47]. In addition, genes involved in xenobiotic metabolism and coagulation cascades are significantly enriched in liver metastasis [48, 49], indicating a strong association between the activation of xenobiotic metabolism and coagulation pathways and the metastatic phenotype.

We also identified specific ligand‐receptor interactions between SCGB3A1‐Epi, KLK10‐Epi, and Fib‐11 in metastatic cancers. In breast cancer liver metastasis, significant interactions were observed between CD74‐APP receptors in SCGB3A1‐Epi and Fib‐11. High expression levels of Fib‐11 and CD74 were associated with prolonged survival in breast cancer patients. Disruption of the APP‐CD74 axis enhances the phagocytic activity of tumor‐associated macrophages in glioblastoma multiforme [50]. CD74 has been implicated as a pro‐oncogenic molecule that promotes invasion and metastasis of triple‐negative breast cancer [51], and some studies have suggested that high CD74 expression is associated with better prognosis of breast cancer patients, positively correlating with immune cell infiltration and PD1 expression [52]. Our findings support the latter conclusion. For PDAC, interactions between KLK10‐Epi and Fib‐11 may be mediated through SPP1‐CD44 receptor pairs, with elevated SPP1 and CD44 expression associated with poorer survival outcomes. Li et al. demonstrated that TME factors promote cancer stemness and metastasis via the SPP1‐CD44 axis, suggesting that targeting this axis could improve PDAC treatment efficacy [53]. Our results align with these findings, further validating KLK10‐Epi as a significant driver of PDAC liver metastasis.

While our bioinformatics approach provides valuable insights, it has limitations, including the lack of direct experimental validation. Future work should include in vitro and in vivo experiments to validate the functional roles of SCGB3A1‐Epi and KLK10‐Epi in liver metastasis. Additionally, exploring the clinical relevance of these findings through prospective studies could enhance their translational potential.

In summary, our study identified SCGB3A1‐Epi and KLK10‐Epi as highly malignant cell subtypes driving liver metastasis in breast cancer and PDAC. These subtypes engage in specific ligand‐receptor interactions with Fib‐11 through CD74‐APP and SPP1‐CD44 pathways, promoting liver metastasis. Future research should focus on elucidating the underlying molecular mechanisms and exploring the therapeutic potential of targeting these interactions to reduce metastasis and improve patient survival.

## Author Contributions

**Zixue Xuan:** conceptualization, data curation, formal analysis, funding acquisition, writing – original draft. **Zhongxiu Wu:** formal analysis, writing – original draft. **Lei Cheng:** formal analysis, writing – original draft. **Jinying Jiang:** visualization, writing – review and editing. **Yuan Zhang:** validation, funding acquisition, writing – review and editing. **Yuxuan Xia:** conceptualization, supervision, writing – review and editing. All the authors have read and approved the final manuscript.

## Ethics Statement

The data utilized in this study were sourced from publicly accessible databases, ensuring that no ethical issues were triggered as the data were already de‐identified and available for research purposes without requiring additional consent.

## Conflicts of Interest

The authors declare no conflicts of interest.

## Data Availability

The data that support the findings of this study are available from the corresponding author upon reasonable request.
